# Correction

**DOI:** 10.1080/22221751.2026.2712857

**Published:** 2026-08-05

**Article title:** *A new vaccination regimen using adenovirus-vectored vaccine confers effective protection against African swine fever virus in swine*

**Authors:** Liu W, Li H, Liu B, Lv, T, Yang C, Chen S, Feng L, Lai L, Duan Z, Chen X, Li P, Guan S & Chen L.

**Journal:** *Emerging Microbes & Infections*

**Bibliometrics:** Volume 12, Issue 1

**DOI:** https://doi.org/10.1080/22221751.2023.2233643

When this article was first published online, Figure 7A contained a typographical error. The dose in the figure was incorrectly labelled as “**vp/mouse**” and has now been corrected to “**vp/swine.**” Additionally, the authors have revised the legend for **Figure 7D** to provide a more detailed description. The revised legend for Figure 7 and the revised is as follows:

**Figure 7.**
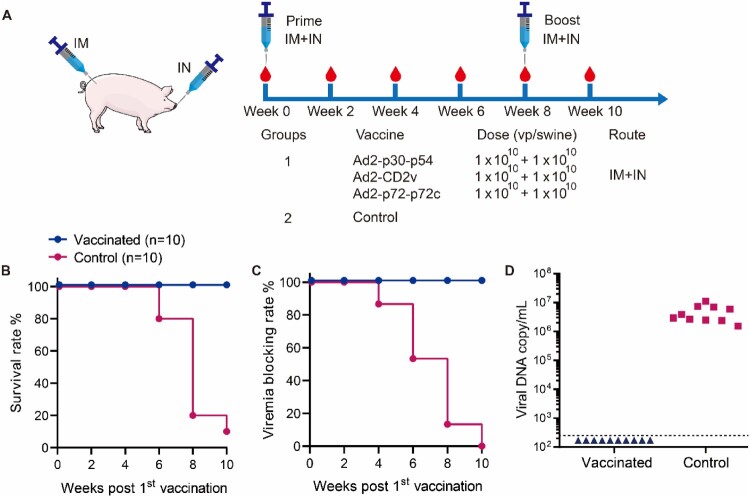
**Vaccine efficacy of Ad2-ASFV in commercial pigs. (A)** Schematic of the prime-boost vaccination scheme in swine. Swine received a second dose of the same vaccine 56 days later. The red blood drop symbols indicate the time points when the blood samples were collected. **(B)** Survival rates of pigs. **(C)** Viremia blocking rates of pigs. **(**D) Viral DNA copies in blood samples of pigs. Viral p72 gene copies were detected using qPCR in blood samples collected from ten vaccinated pigs at the week 10 endpoint, nine pigs in the control group on the day they died and one pig in the control group that survived at the week 10 endpoint. The lower limit of detection is indicated by the dashed lines.

